# Supplementary material for: Direct mailing of HPV self-sampling kits to women aged 50–65 non-participating in cervical screening in the Czech Republic
Source: Eur J Public Health. 2024 Jan 15;34(2):361–7. doi: 10.1093/eurpub/ckad229 (PMC10990530; doi:10.1093/eurpub/ckad229)
Supplement: ckad229_Supplementary_Data [file ckad229_supplementary_data.docx]

**Supplementary Table 1: Characteristics of participants by study group and type of examination**

| **Characteristics** | **Returned and evaluated HPVssk** HPVssk group | **Examined by  a gynaecologist**  HPVssk group | | **Examined by  a gynaecologist**  Control group | **Comparison**^c^ | |  |
| --- | --- | --- | --- | --- | --- | --- | --- |
|  | **No. (%)** | | **No. (%)** | **No. (%)** | | **p-value** | |
| Age group |  | |  |  | |  | |
| 50-59 | 36 (61.0%) | | 26 (54.2%) | 23 (60.5%) | | 0.745 | |
| 60 and over | 23 (39.0%) | | 22 (45.8%) | 15 (39.5%) | |  | |
| No. of invitations^a^ |  | |  |  | |  | |
| 1-2 | 14 (23.7%) | | 29 (60.4%) | 19 (50.0%) | | <0.001 | |
| 3 and more | 45 (76.3%) | | 19 (39.6%) | 19 (50.0%) | |  | |
| Letter variant^b^ |  | |  |  | |  | |
| C, C and M, C and K | 33 (55.9%) | | 31 (64.6%) | 28 (73.7%) | | 0.204 | |
| C and M and K | 26 (44.1%) | | 17 (35.4%) | 10 (26.3%) | |  | |

a represents how many times a woman has received an invitation

b describes what screening programmes the invited women did not attend; C – cervical cancer screening, M – breast cancer screening, K – colorectal cancer screening

c comparison were made using the Pearson chi-square test
